# Prevalence and Outcomes of Depression After Bariatric Surgery: A Systematic Review and Meta-Analysis

**DOI:** 10.7759/cureus.25651

**Published:** 2022-06-04

**Authors:** Rayyan A Alyahya, Muhaid A Alnujaidi

**Affiliations:** 1 Pediatrics and Neonatology, Prince Sultan Military Medical City, Riyadh, SAU; 2 General Surgery, Prince Mohammed Bin Abdulaziz Hospital, Riyadh, SAU

**Keywords:** surgery, outcomes, prevalence, bariatric surgery, depression

## Abstract

Surgeons often focus on weight loss and improvement of obesity-related conditions as a primary outcome after bariatric surgery. However, the success of bariatric surgery also relies on the improvement of mental health status. Therefore, the current meta-analysis was carried out to reveal the prevalence of depressive symptoms and their subsequent impact on bariatric surgery outcomes. This study was performed following the Preferred Reporting Items for Systematic Reviews and Meta-Analyses (PRISMA) checklist and the recommendation of Cochrane Collaboration. All clinical studies reporting the prevalence and/or the outcomes of depression after bariatric surgery were included in the current meta-analysis. This meta-analysis encompassed 33 articles, including a total of 101,223 patients. The prevalence of post-bariatric surgery depression was 15.3% (95% confidence intervals {CI}: 15.0-15.5%, p<0.001) among which severe, moderate, and minimal depression accounted for 1.9% (95% CI: 1.5-2.4%, p<0.001), 5.1% (95% CI: 4.4-5.8%, p<0.001), and 64.9% (95% CI: 63.3-66.5%, p<0.001), respectively. Depression is negatively correlated with weight loss (correlation -0.135; 95% CI: -0.176 to 0.093; p<0.001) and positively correlated with eating disorder (correlation 0.164; 95% CI: 0.079-0.248; p<0.001). The prevalence of post-bariatric surgery depression is relatively high reaching up to 64.9%, with almost one in five patients affected by it. Depression is associated with weight regain, eating disorders, and quality of life.

## Introduction and background

Obesity is a complex health problem with a growing incidence worldwide [1]. To date, approximately 1.9 billion and 610 million adults are considered overweight and obese, respectively, representing nearly 39% of the general population [2,3]. Obesity negatively impacts all physical and mental aspects of the body. It leads to cardiovascular insufficiency, metabolic syndrome, hepatobiliary diseases, respiratory disorders, osteoarthritis, infertility, and cancer. Besides that, obesity might be associated with anxiety, low self-esteem, depression, and impaired quality of life (QoL) [4-6]. These significant consequences limit the patients’ performance, decrease their chances of getting a job due to physical appearance, increase their absenteeism frequency, and enhance isolation and addiction risks [7,8]. Obese patients are nearly 55% more vulnerable to experience depressive symptoms than the non-obese population. Furthermore, approximately 45% of bariatric surgery seekers present with depression [9,10].

A number of modalities have been proposed for treatment of obesity. Bariatric surgery is considered the safest and the most effective procedure for weight reduction, which reduces obesity-related comorbidities and improves survival [11-13]. Surgeons often focus on weight loss and improvement of obesity-related conditions as a primary outcome after bariatric surgery [14]. However, it has been widely accepted that success after bariatric surgery depends not only on weight loss but also on the improvement of mental health status [9]. While most patients show improved psychological state after bariatric surgery, a considerable proportion experience persistent psychological concerns and even worsening manifestations [15].

Patients undergoing bariatric surgery are associated with a fourfold increase in the risk of attempted suicide as compared to the general community [16,17]. Assessment of post-bariatric psychological outcomes is critical to identify morbidly obese patients who require further supportive treatment [18]. A deeper insight into the mental state of the patients undergoing bariatric surgery can contribute to a more comprehensive understanding of and identify patients at a higher risk of post-operative depression [19].

The prevalence and subsequent outcomes of depression after bariatric surgery are still unclear in the literature [20-22]. Previous investigations have focused mainly on pre-operative depression, and little is known about the impact of depression after undergoing bariatric surgery [23]. Identifying the relationship between depression and success of bariatric surgery is critical, considering that inadequate weight loss after surgery might lead to the re-emergence of obesity and its associated complications, thereby impairing the patient’s QoL [24]. Furthermore, this knowledge will help healthcare providers to identify patients at risk and employ timely and appropriate management of depression after bariatric surgery to prevent its potential long-term consequences. Therefore, the current systematic review and meta-analysis were carried out to reveal the prevalence of depressive symptoms and their subsequent effects on the short-term and long-term outcomes of bariatric surgery.

## Review

Methods

This systematic review and meta-analysis was carried out following the Preferred Reporting Items for Systematic Reviews and Meta-Analysis (PRISMA) guidelines [25]. An extensive systematic review of literature up to October 17, 2020, was implemented using the following databases: PubMed, Google Scholar, Web of Science (ISI), Scopus, SIGLE, Virtual Health Library (VHL), NYAM, ClinicalTrials, metaRegister of Controlled Trials (mRCT), Embase, and WHO International Clinical Trials Registry Platform. No restrictions were set in terms of patients’ age, sex, ethnicity, language, race, or place. The following keywords were used in every possible combination: “bariatric,” “sleeve,” “gastric bypass,” “gastric band,” “duodenal switch,” “depression,” and “depressive.” A further manual search was performed to comprehend all retrieved studies’ references to distinguish all additional relevant articles that were not indexed. The cross-referencing method was carried out until no other relevant article was detected.

Study selection

All clinical studies that reported the prevalence and/or the outcomes of depression after bariatric surgery were included in the current meta-analysis. This includes studies comparing the outcomes of depressed and non-depressed patients after the surgery and also single-arm studies that reported the association between depression and bariatric surgery outcomes. There were no restrictions on the patients’ age, sex, race, or place. In contrast, studies that did not report an association between depression and surgery outcomes were excluded. Furthermore, studies in which data could not be extracted, such as guidelines, review articles, animal studies, case reports, comments, letters, editorials, posters, and book chapters, were excluded without adding any restriction on langauge. The screening process of the title, abstract, and full text was performed independently to reveal potentially relevant articles that met the inclusion criteria. Discussions were carried out to resolve contradictions among reviewers.

Data extraction and quality assessment

The following data were extracted from the finalized included articles: study characteristics (the title of the included study, the second name of the first author, year of publication, study design, study period, study region, and sample size), patients’ demographic characteristics (age, sex, weight, height, body mass index {BMI}, occupation, comorbidities, family history of psychiatric illness, and pre-operative psychological status), bariatric surgery-related data (the type of surgery, initial weight loss, and intra-operative and post-operative complications), post-operative psychological status (depression screening tools, duration of the current episode, number of depressed patients, number of suicide attempts, QoL scores, and post-operative depression score), and psychological outcomes (number of depressed patients, the correlation between post-operative depression and eating disorders, weight loss, body image, regained BMI, BMI loss, and mental and physical components of QoL).

The quality of the observational studies was assessed using the National Institute of Health quality assessment tool [26]. The studies were grouped based on the quality assessment into good (quality score >65%), fair (quality score 30-65%), and bad (quality score <30%). If the parameter was controlled, the domain was considered “yes” and vice versa.

Statistical analysis

The prevalence of depression was estimated by calculating the event rate with 95% confidence intervals (CIs) for each study, followed by pooling the effect sizes of all studies to estimate the summary proportion with 95% CIs. The summary correlation and 95% CIs were computed by pooling the correlation and sample size of each relevant article. The fixed-effect model was implemented when a fixed population effect size was assumed; otherwise, the random-effects model was used. Statistical heterogeneity was appreciated using Higgins I^2^ statistic, at the value of >50%, and the Cochran's Q (chi-square test), at the value of p<0.10 [27]. To account for heterogeneity, the random-effects model was employed. Publication bias was assumed in the presence of an asymmetrical funnel plot and based on Egger’s regression test (p<0.10). Herein, the trim and fill method of Duval and Tweedie was used [28]. Subgroup analysis was conducted based on the severity of depressive manifestations. Data analysis was performed using the Review Manager version 5.3 (Copenhagen, Denmark: The Nordic Cochrane Centre, The Cochrane Collaboration) and Comprehensive Meta-Analysis software version 2 [29,30]. The significant difference was established at the value of p<0.05.

Results

**Figure 1:**
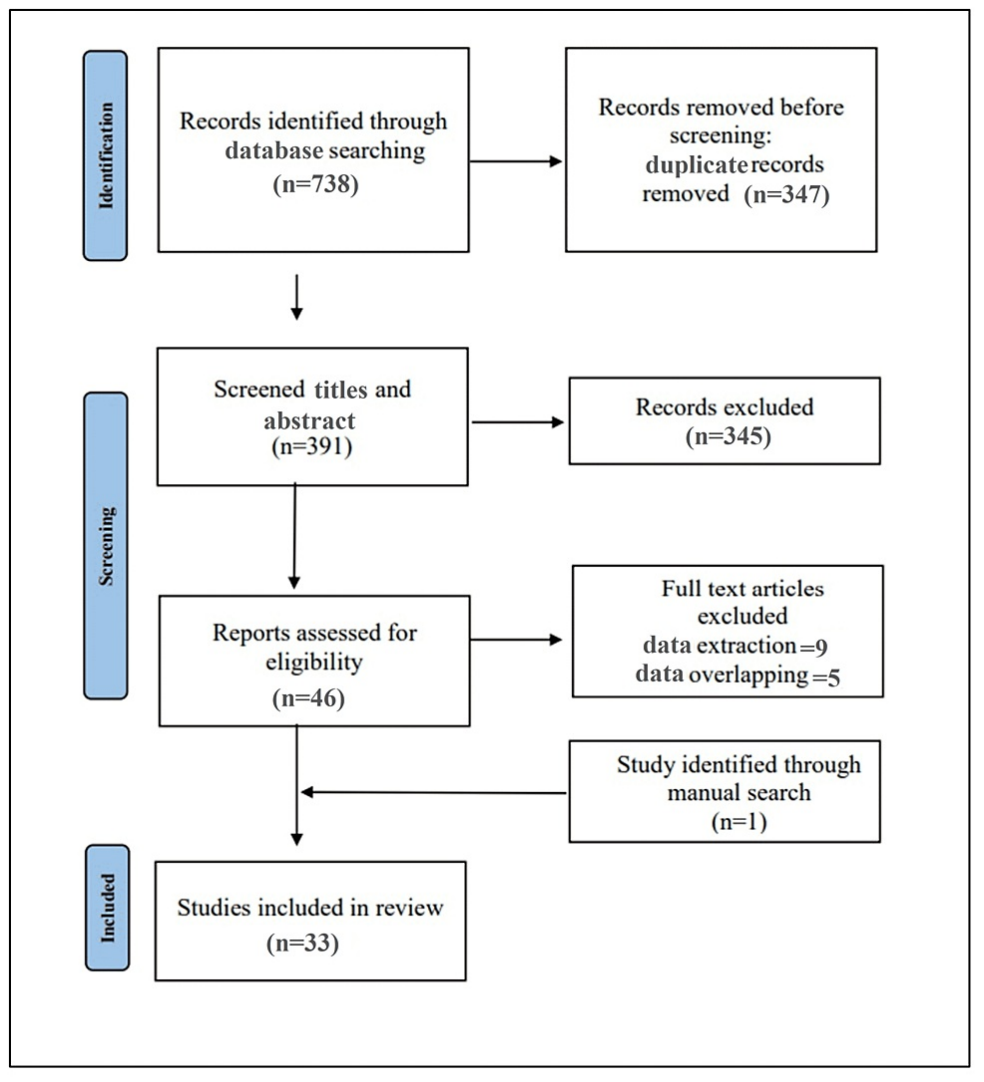
An illustration of the process of literature search.

A comprehensive systematic literature search yielded a total of 738 articles. Using EndNote X9 (London, UK: Clarivate), 347 duplicates were removed, yielding 391 articles eligible for title and abstract screening. Of these studies, 46 articles were suitable for full-text screening, and 37 articles were included for data extraction. Out of them, five studies were excluded due to overlapping data. Herein, 32 articles were included for systematic review and meta-analysis in addition to one study identified through manual search. A flow diagram illustrating the process of literature search is shown in Figure 1.

Study characteristics

**Figure 2:**
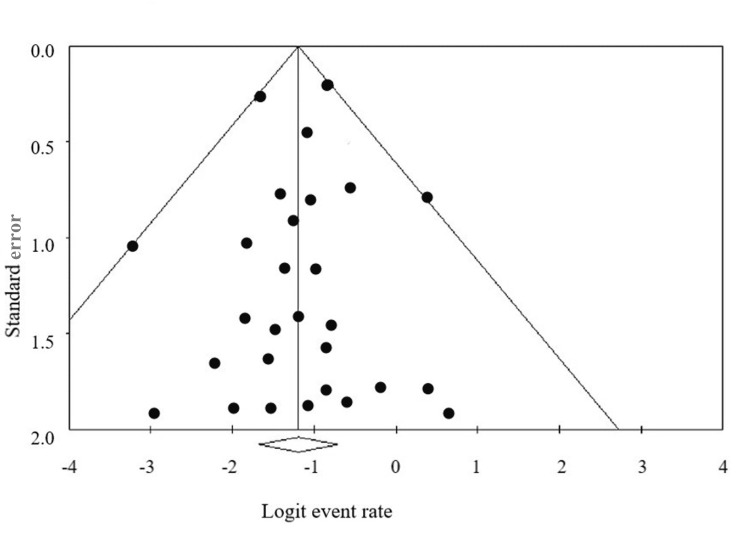
Funnel plot to assess publication bias across prevalence studies.

This meta-analysis encompassed 33 articles, including a total of 101,223 patients. There were 76.33% (27,674/36,282) females. At the baseline, the mean age of the included patients ranged from 32.2 to 47.61 years. The mean BMI ranged from 42.02 to 51.8 kg/m^2^, and the average pre-operative depression score ranged from 7.7 to 20.2. The mean follow-up period ranged from six to 45.6 months. Of the included studies, three studies showed fair quality, and the remaining articles were of good quality [31-33]. The Funnel plot was found to be symmetrical, which indicates no publication bias (Table 1, Figure 2).

**Table 1:** Demographic characteristics of the included studies. *Range **Median and range ***Mean and range BDE: Beck Depression Inventory; EDE-Q: Eating Disorder Examination-Questionnaire; BSQ: Body Shape Questionnaire, HADS: Hospital Anxiety and Depression Scale; HAM-D Scale: Hamilton Depression Scale; SF-36: Medical Outcomes Study Short Form-36 Health Survey; IPAQ: International Physical Activity Questionnaire; PHQ-9: Patient Health Questionnaire-9; RSES: Rosenberg Self-Esteem Scale; CESD: Center for Epidemiologic Studies Short Depression Scale; NR: non-reported

S. no.	Study ID	Study region	Study design	Study period	Sample size (number)	Gender (female) (number)	Age (mean± SD)	Type of Procedure				BMI (Mean± SD)	Psychological Assessment	Follow-up Period	Quality Assessment	
								Gastric banding (number)	Roux-en-Y gastric bypass (number)	Gastric Sleeve (Number)	Duodenal Switch (Number)				%	Decision
1.	Brandão et al., 2016 [34]	Portugal	Retrospective observational and cross-sectional study	January 2009 and June 2013	75	64	(63-64)*	47	19	9	0	44.75 (34.53-59.82)**	BDI, EDE-Q, BSQ	(18-46) month*	78%	Good
2.	Sousa et al., 2014 [35]	Portugal	Retrospective	NR	52	43	44.04 (10.87)	38	5	9	0	NR	BDI	(22-132) month*	75%	Good
3.	Andersen et al., 2010 [36]	Norway	Prospective cohort study	NR	50	28	37.9±7.9	0	0	0	50	NR	HADS>8	2 years	80%	Good
4.	de Zwaan et al., 2011 [37]	Germany	Prospective cohort study	NR	107	75	37.5±9.7	76	31	0	0	49.4±7.4	DSM-IV	(24-36) month*	80%	Good
5.	Freire et al., 2020 [38]	Brazil	Retrospective	1999 and 2004	96	75	40.2± 10.1	0	96	0	0	50±8.2	BDI	2 years	78%	Good
6.	Pinto et al., 2017 [39]	Brazil	Prospective cohort study	NR	60	51	34.7±9.2	NR	NR	NR	NR	46.04±7.52	BDI-SF>4	NR	67%	Good
7.	Nijamkin et al., 2013 [40]	USA	Prospective cohort study	NR	144	120	44.5±13.5	0	144	0	0	35.95±5.9	BDI-II	18 months	76%	Good
8.	Mitchell et al., 2014 [41]	USA	Randomized clinical trial	February 2006 and February 2009	2,146	1,685	46 (37.54)	539	1,507	NR	NR	45.9 (41.8-51.4)**	BDI	2 years	87%	Good
9.	Jans et al., 2018 [42]	Flemish	Randomized clinical trial	December 2012 until March 2016	54	NR	29.4±4.3	2	45	2	0	28.1±5.1	Dutch pregnancy-validated Edinburgh Depression Scale	45.6±29.9	85%	Good
10	Alabi et al., 2018 [43]	Mexico	Retrospective	January 2015 and January 2016	73	56	38.1±9.1	NR	NR	NR	NR	38.8±3.8	BDI-II	12 months	83%	Good
11	Nicolau et al., 2017 [44]	Spain	Retrospective	NR	60	47	45.5±9.4	NR	NR	NR	NR	48.4±7.6	BDI-II, SF-36 Health Survey Spanish version	46.48±18.1	75%	Good
12	Bressan et al., 2019 [33]	Brazil	Cross-sectional study	2015 and 2016	71	54	39.8±10.3	NR	NR	NR	NR	NR	BDI-II, Rosenberg Self-Esteem Scale	NR	55%	Fair
13.	Yuan et al., 2019 [45]	USA	Retrospective claims data from Aetna	2008 and 2016	64,090	NR	46.19±13.59	NR	NR	NR	NR	NR	BDI-II	748 days	88%	Good
14.	Osterhues et al., 2017 [10]	Germany	Randomized clinical trial	September 2015 and March 2016	103	80	43.30±11.69	NR	NR	NR	NR	NR	HADS ≥8	NR	68%	Good
15.	Booth et al., 2015 [46]	UK	A controlled interrupted time-series	January 1, 2000, to April 30, 2012	3,045	2,406	45.9±10.2	NR	NR	NR	NR	44±8.3	NR	(2-3)* years	78%	Good
16.	Elwan et al., 2014 [47]	Egypt	Prospective cohort study	January 2012 and June 2014	30	22	33.80±9.61	0	0	15	0	46.0±1.55	HAM-D Scale	19.56±6.92 month	81%	Good
17.	Lu et al., 2018 [48]	Taiwan	Retrospective from National Health Insurance Research Database of Taiwan	2001 to 2009	2,102	1,425	32.2±9.8	NR	NR	NR	NR	NR	NR	NR	68%	Good
18.	Timofte et al., 2018 [49]	Romania	Prospective cohort study	NR	7	3	NR	0	0	7	0	NR	Montgomery-Asberg Depression Rating Scale	NR	71%	Good
19.	Susmallian et al., 2019 [32]	Israel	Prospective, midterm follow-up study	January 2013 to December 2014	253	NR	41.65±11.05	0	0	253	0	42.02±5.03	NR	NR	59%	Fair
20.	Sivas et al., 2020 [50]	Turkey	Prospective cohort study	January 2016 and May 2017	27	23	37.1±10.4	NR	NR	NR	NR	46.2±5.2	BDI-II, IPAQ	NR	69%	Good
21.	Sait et al., 2019 [51]	Saudi Arabia	Cross-sectional study	July 2013 and July 2017	214	184	NR	0	32	177	0	NR	PHQ-9	NR	72%	Good
22.	Porcu et al., 2011 [52]	Brazil	Prospective cohort study	NR	50	NR	NR	NR	NR	NR	NR	NR	BDI, the Hospital Scale of Anxiety and Depression (I-TAD)	NR	45%	Fair
23.	White et al., 2015 [16]	USA	Prospective cohort study	NR	357	NR	43.7±10	NR	NR	NR	NR	51.2±8.3	BDI-II, EDEQ, Short Form-36 Health Survey	24 months	73%	Good
24.	Martens et al., 2020 [53]	USA	Prospective cohort study	2015-2017	1,991	1,573	47.61±11.63	0	324	1,667	0	47.42±8.04	Patient Health Questionnaire	NR	69%	Good
25.	Lu et al., 2019 [54]	USA	Prospective cohort study	NR	103	103	44.1±11.7	NR	NR	NR	NR	45.3±6.2	CESD short scale	NR	71%	Good
26.	Barzin et al., 2020 [55]	Iran	Prospective cohort study	March 2014 to March 2016	685	581	38.7±10.9	0	242	443	0	45.1±6.0	BDI-II	NR	75%	Good
27.	Lagerros et al., 2017 [56]	Sweden	Retrospective from National Health Insurance Research Database of Taiwan	2008 and 2012	22,539	16,961	41.3	0	22,539	0	0	NR	ICD-diagnoses F32-F33 forms	546 (2-730) days***	88%	Good
28.	Méa et al., 2017 [57]	Brazil	Cross-sectional observational study	NR	20	11	NR	NR	NR	NR	NR	NR	BDI-II	NR	71%	Good
29.	Matini et al., 2014 [58]	Iran	Prospective observational study	May 2012 to May 2013	67	55	36.8±8.5	NR	NR	NR	NR	48.8±4.7	HDRS	6 months	67%	Good
30.	Grilo et al., 2007 [59]	USA	Prospective cohort study	NR	137	NR	42.3±10.2	0	137	0	0	51.8±7.9	BDI-II, Short Form-36 Health Survey	12 months	75%	Good
31.	Smith et al., 2020 [60]	USA	Prospective cohort study	2006-2009	2,308	1,816	45.5±11.4	0	2,308	0	0	NR	BDI	3 years	67%	Good
33.	Ivezaj et al., 2014 [61]	USA	Prospective cohort study	NR	107	94	42.7±10.5	0	107	0	0	51.7±7.8	BDI, EDE-Q, SF-36, and RSES	12 months	75%	Good

Prevalence of post-bariatric surgery depression

**Figure 3:**
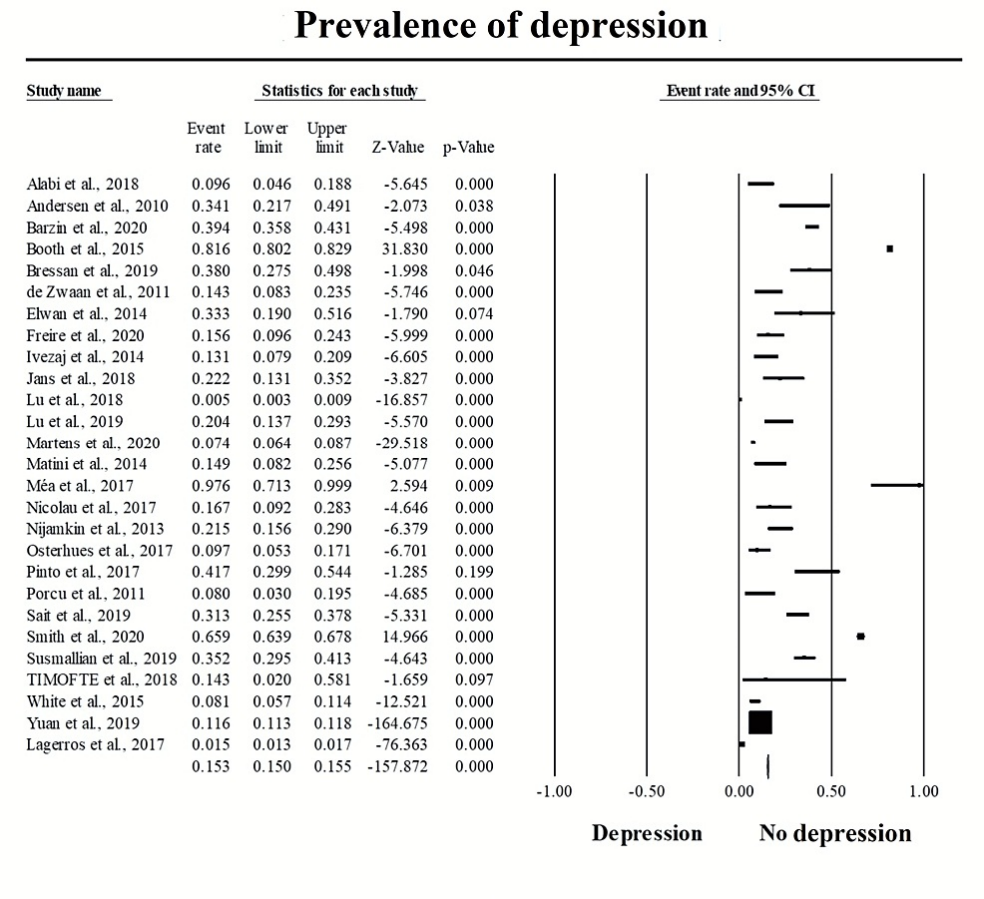
Pooling of the prevalence of post-bariatric depression with subgroup analysis. Pooling the data revealed a prevalence rate of 15.3% (95% CI: 15-15.5%, p<0.001).

**Figure 4:**
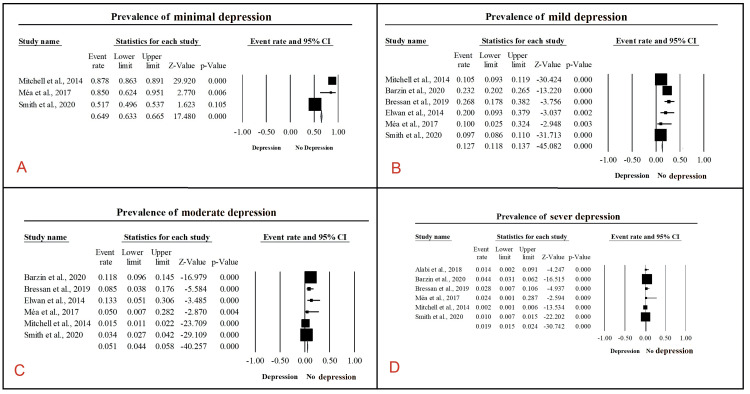
Prevalence of post-bariatric surgery depression. The image shows the prevalence rate of (A) minimal depression: 64.9% (95% CI: 63.3-66.5%, p<0.001); (B) mild depression: 12.7% (95% CI: 11.8-13.7%, p<0.001); (C) moderate depression: 5.1% (95% CI: 4.4-5.8%, p<0.001); (D) severe depression: 1.9% (95% CI, 1.5-2.4%, p<0.001).

A total of 27 articles, including 98,757 patients, reported the prevalence of post-bariatric depression. Pooling the data revealed a prevalence rate of 15.3% (95% CI: 15.0-15.5%, p<0.001) (Figure 3) [10,16,32,33,36-40,42-49,51-58,60,61]. Subgroup analysis among patients with depression revealed that prevalence of severe depression was 1.9% (95% CI: 1.5-2.4%, p<0.001). The prevalence of moderate depression was 5.1% (95% CI: 4.4-5.8%, p<0.001), whereas the prevalence of mild and minimal depression was 12.7% (95% CI: 11.8-13.7%, p<0.001), and 64.9% (95% CI: 63.3-66.5%, p<0.001) (Figure 4, panels A-D) [33,41,43,47,55,57,60].

Impact of depression on bariatric surgery outcomes

Weight Loss and BMI

**Figure 5:**
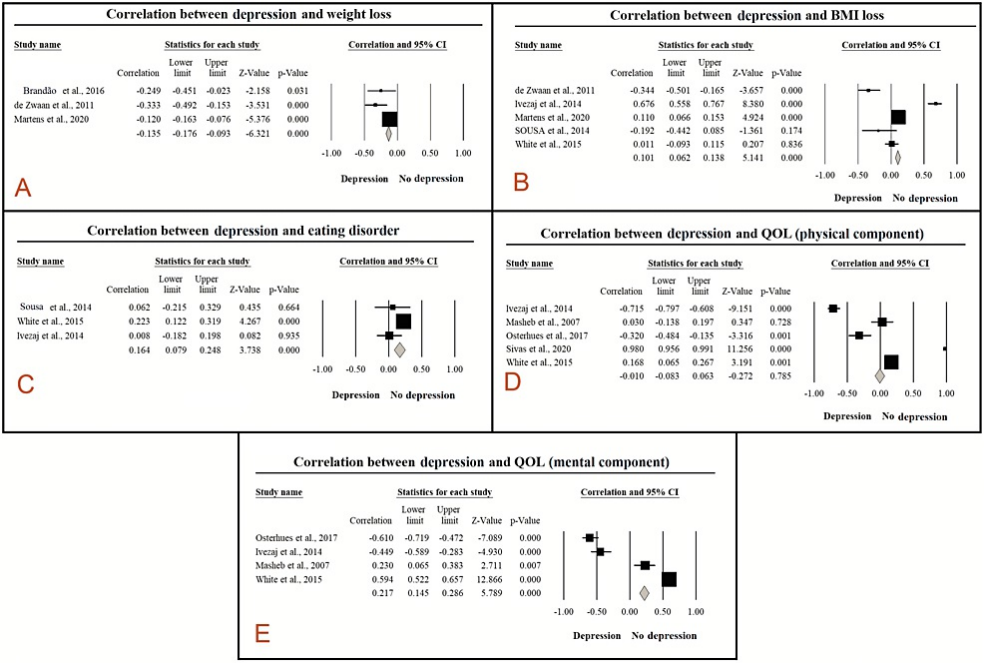
Correlation between depression and surgery outcomes. (A) Weight loss, correlation: -0.135, 95% CI: -0.176 to -0.093, p<0.001; (B) BMI loss, correlation: 0.011, 95% CI: -0.093 to -0.115, p=0.836; (C) eating disorder, correlation: 0.164, 95% CI: 0.079-0.248, p<0.001; (D) quality of life (physical component), correlation: -0.010, 95% CI: -0.083 to 0.063, p=0.785; and (E) quality of life (mental component), correlation: 0.217, 95% CI: 0.145-0.286, p<0.001.

The association between post-bariatric depression and weight loss was reported in three articles, including 2,173 patients. In the random-effects model (p=0.048, I^2^=67%), there was a statistically significant negative association between post-operative depression and weight loss (correlation -0.135; 95% CI: -0.176 to -0.093; p<0.001). Conversely, there was no statistically significant association between post-bariatric surgery depression and BMI (correlation 0.011; 95% CI: -0.093 to -0.115; p=0.836) (Figure 5, panels A and B) [16,34,35,37,53,61].

Eating Disorder

Three studies, including 516 cases, evaluated the correlation between post-bariatric depressive manifestations and eating disorders. There was a statistically significant positive association (correlation 0.164; 95% CI: 0.079-0.248; p<0.001) between post-operative depression and eating disorders in the random-effects model (p=0.109, I^2^=54%) (Figure 5, panel C) [16,35,61].

Quality of Life

The impact of post-bariatric surgery depression on the mental component of the QoL was assessed among 704 patients from four studies. In the random-effects model (p<0.001, I^2^=98%), pooling the effect sizes revealed a statistically significant association between post-bariatric depressive manifestations and mental component of QoL (correlation 0.217; 95% CI: 0.145-0.286; p<0.001). However, there was no statistically significant association between post-operative depression and the physical component of QoL (correlation -0.010; 95% CI: -0.083 to 0.063; p=0.785) (Figure 5, panels D and E) [10,16,50,59,61].

Discussion

Bariatric surgery procedures are associated with clinically significant weight reduction, improvements in overweight-related comorbidities, and prolonged life expectancy [62]. It positively affects the patients’ physical and mental aspects of life, including daily activities, social relationships, body image, eating behavior, sexual life, and mental health. There is relative individual variation in the weight reduction after surgery, and some patients might experience worsening of their psychological health status [63,64]. Despite the growing body of evidence related to bariatric surgery outcomes, there is limited literature regarding the impact of the surgery on psychological outcomes [15,65]. Therefore, this meta-analysis was performed to assess the prevalence of post-bariatric depressive manifestations and evaluate how these manifestations affect surgery outcomes.

Our systematic review and meta-analysis revealed that approximately one in every five patients who underwent bariatric surgery would experience depression at any interval within three years after surgery. The proportion of patients at risk to develop minimal depression after bariatric surgery was considerably high (more than 50% of bariatric surgery seekers). These findings are comparable with Courcoulas et al. who reported a decline of mild depression manifestations from 28% to 9.8% six months after surgery, followed by new rise to 12.2% and 15.6% in the second and third years after surgery, respectively [66]. In the short-term period, post-bariatric depressive manifestations might not have a significant impact on weight regain. Instead, initial weight reduction is related mainly to the bariatric surgery-induced metabolic changes rather than behavioral or psychological factors.

Most of the weight reduction occurs during the first year after bariatric surgery. This period of rapid weight loss is rewarding for patients to lose more weight. However, after this period, the weight loss plateaus, requiring patients to adopt overly restrictive and long-term nutritional and behavioral modifications to lose any additional weight [67]. The resultant loose skin and plateauing of body weight after rapid weight loss are associated with a high risk of body dissatisfaction [68]. These situations are accompanied by unrealistic expectations regarding rapid weight loss and body contouring, which puts the patients under more stress [69]. Patients at a higher risk of post-bariatric depression should be subjected to close monitoring. This includes exhaustive pre-operative assessment of depression and psychological disorders, along with employing timely and effective anti-depressive interventions [70]. This could enhance the effectiveness of the surgery, amplify weight reduction after surgery, and improve the long-term QoL. However, further studies with an adequate long-term period are required for comprehensively understanding the trajectory of depressive manifestations and weight regain after bariatric surgeries.

Identifying factors associated with long-term suboptimal weight loss in patients seeking bariatric surgery is of great importance to minimize the risk of revision surgery, psychological illness, and costs associated with suboptimal weight reduction [71,72]. In this meta-analysis, post-bariatric depression was associated with weight regain, eating disorders, and poor QoL. These results reinforce the close association between obesity and depressive manifestations, wherein both conditions could be dependent on each other [73,74]. In this regard, Geerts et al. reported that suboptimal weight loss after bariatric surgery was associated with impulsive eating, eating disorders, and depression [75]. Switzer et al. reported a strong association between rebound weight gain and depressive manifestations after bariatric surgery [76]. In a systematic review, Hindle et al. reported a significant association between early post-operative weight loss, eating adaptation, and later long-term weight loss. However, the evidence regarding the association between early post-operative psychological disturbance and later weight gain was not sufficient and inconstant to reach a definitive conclusion [23].

To the best of our knowledge, this is the first systematic review that gathered the rapidly emerging controversial evidence regarding the prevalence of post-bariatric surgery depression and its subsequent impact on the surgery outcomes. However, some limitations should be acknowledged. The majority of the included articles were of observational design, revealing a potential risk of selection bias. There was significant heterogeneity between the included studies. This heterogeneity might stem from different demographic characteristics, assessment methods, and surgical techniques. Due to the short follow-up periods, the long-term prevalence of depression and its impact on bariatric surgery could not be assessed.

## Conclusions

The prevalence of post-bariatric surgery depression is high. Depression is associated with weight regain, eating disorders, and impaired QoL. The integration of these findings in healthcare protocols can help healthcare providers identify patients at a higher risk of depression and enhance bariatric surgery outcomes by stratifying the patients to the most appropriate and effective treatment in a timely fashion. However, further studies need to be conducted to tackle the limitations of the current meta-analysis.
